# Supplementary material for: Assessing the performance of the Cell Painting assay across different imaging systems
Source: bioRxiv. 2023 Feb 15:2023.02.15.528711. Preprint. [Version 1] doi: 10.1101/2023.02.15.528711 (PMC9949001; doi:10.1101/2023.02.15.528711)
Supplement: Supplement 1 [file NIHPP2023.02.15.528711v1-supplement-1.pdf]

bioRxiv preprint doi: https://doi.org/10.1101/2023.02.15.528711; this version posted February 15, 2023. The copyright holder for this preprint (which was not certified by peer review) is the author/funder, who has granted bioRxiv a license to display the preprint in perpetuity. It is made available under aCC-BY 4.0 International license.

Supplementary Materials

bioRxiv preprint doi: https://doi.org/10.1101/2023.02.15.528711; this version posted February 15, 2023. The copyright holder for this preprint (which was not certified by peer review) is the author/funder, who has granted bioRxiv a license to display the preprint in perpetuity. It is made available under aCC-BY 4.0 International license.

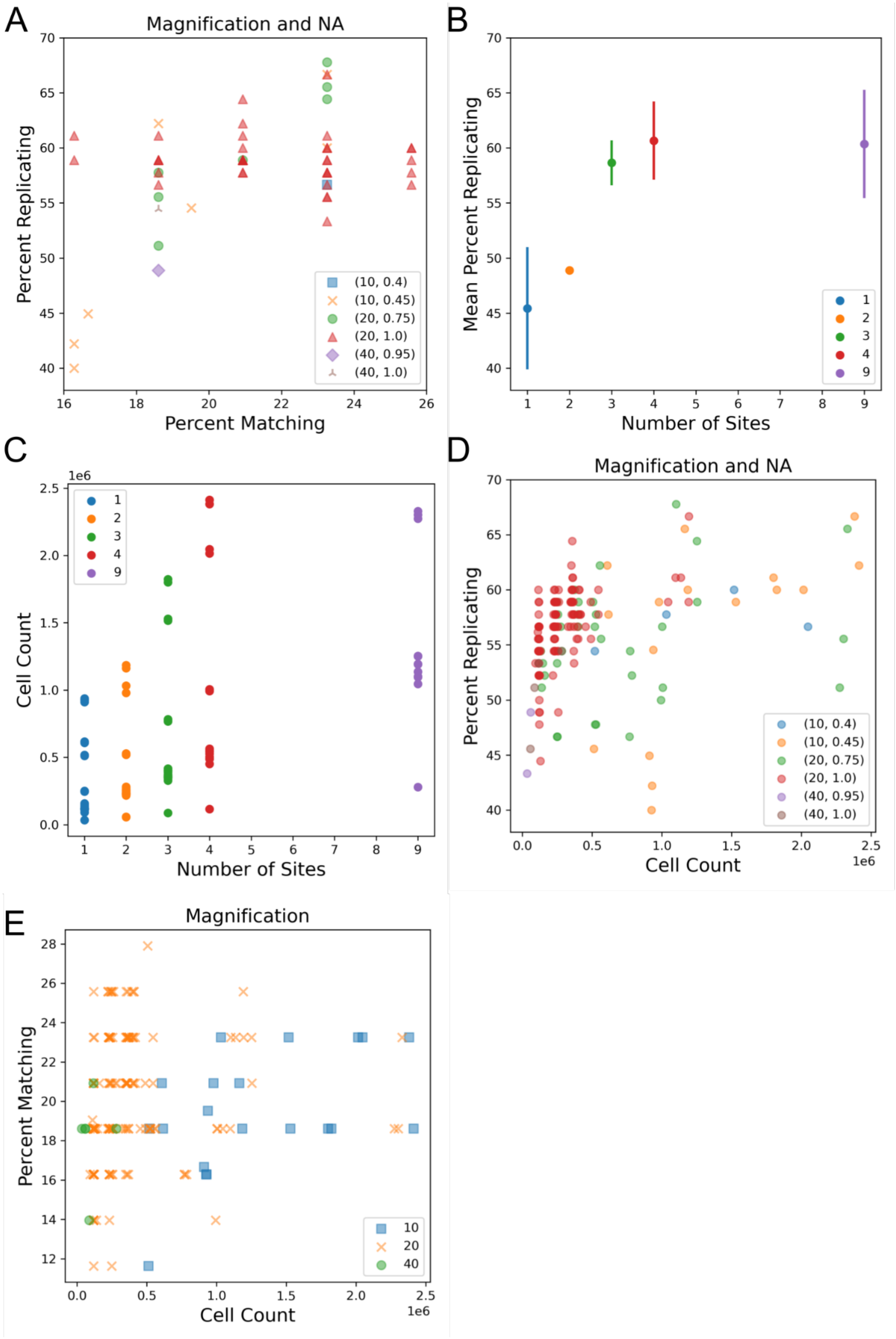

bioRxiv preprint doi: https://doi.org/10.1101/2023.02.15.528711; this version posted February 15, 2023. The copyright holder for this preprint (which was not certified by peer review) is the author/funder, who has granted bioRxiv a license to display the preprint in perpetuity. It is made available under aCC-BY 4.0 International license.

Supplementary figure 1

(A) Comparison of profiles grouped based on magnification and numerical aperture (NA).
(B) Aggregated percent replicating score increases with the number of sites across all profiles.
(C) Cell count increases with the number of sites.
(D) Profiles were grouped based on their magnification and aperture. An increase in cell count is associated with an increase in profile strength. Includes artificially site subsampled data.
(E) Comparison of percent matching versus cell count, grouped by magnification type. Includes artificially site subsampled data.

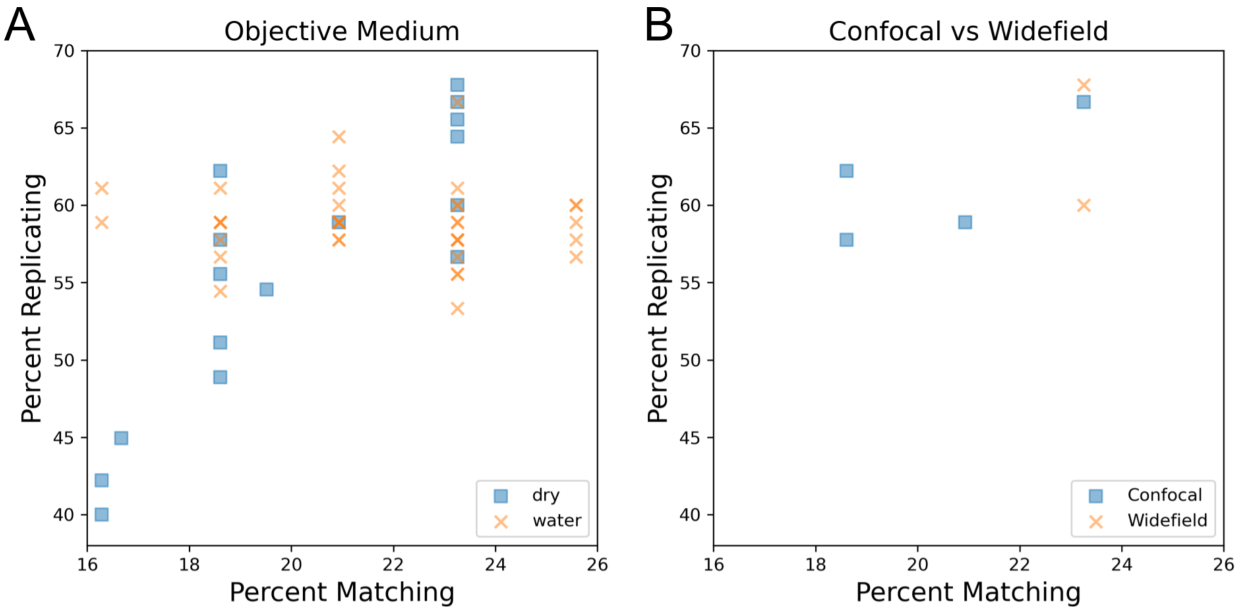

Supplementary figure 2

(A) Objective medium has minimal impact on profile strength.
(B) Comparison of additional confocal vs widefield profiles.

bioRxiv preprint doi: https://doi.org/10.1101/2023.02.15.528711; this version posted February 15, 2023. The copyright holder for this preprint (which was not certified by peer review) is the author/funder, who has granted bioRxiv a license to display the preprint in perpetuity. It is made available under aCC-BY 4.0 International license.

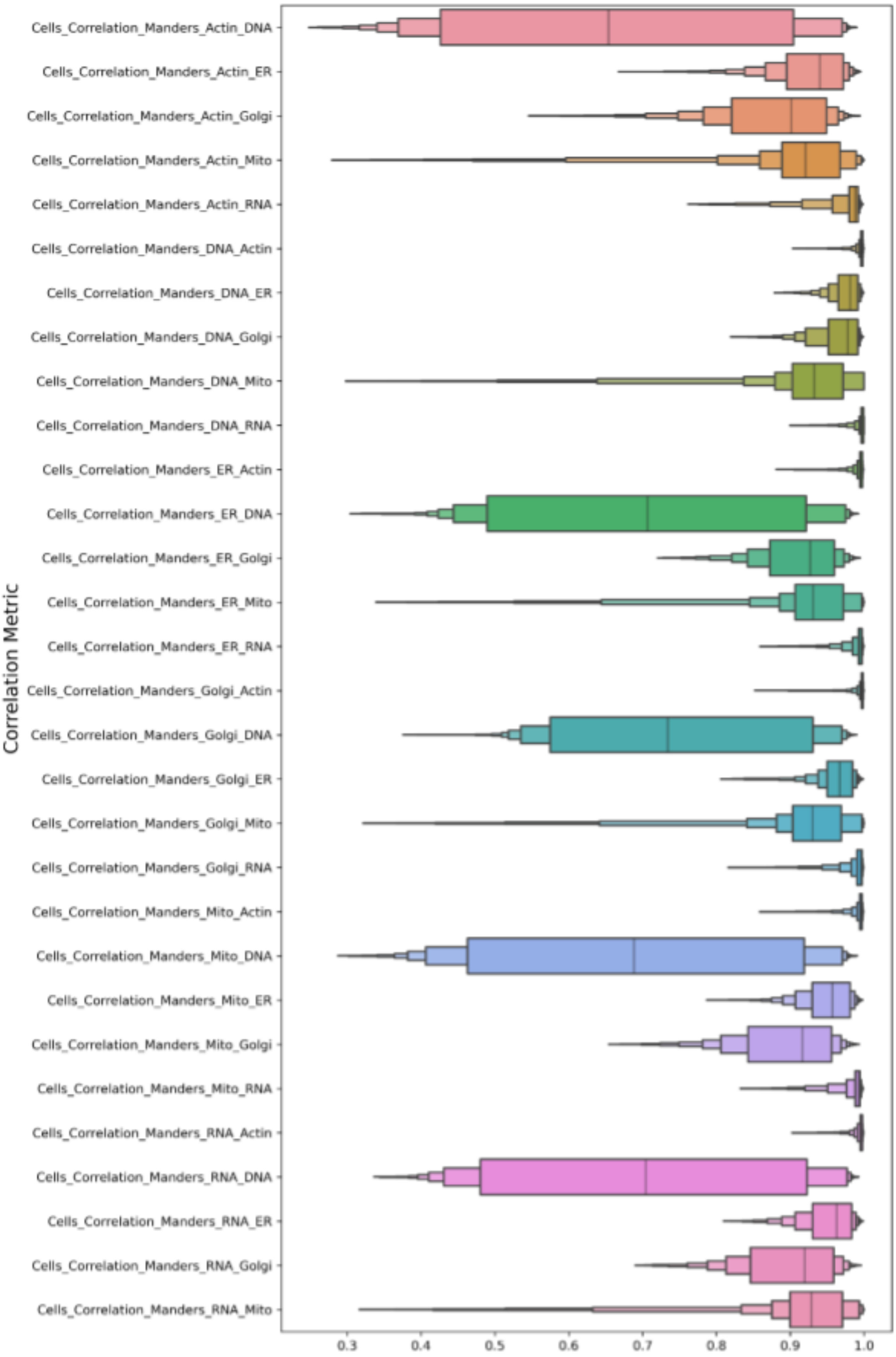

bioRxiv preprint doi: https://doi.org/10.1101/2023.02.15.528711; this version posted February 15, 2023. The copyright holder for this preprint (which was not certified by peer review) is the author/funder, who has granted bioRxiv a license to display the preprint in perpetuity. It is made available under aCC-BY 4.0 International license.

Supplementary figure 3

Unnormalized Manders correlation features within cell objects for all wells of profiles that split AGP into actin and Golgi + plasma membrane channels. High correlation is observed between actin and Golgi (especially above, Golgi_Actin, indicating that actin bleeds through into the Golgi channel), which are spectrally difficult to dissect.

bioRxiv preprint doi: https://doi.org/10.1101/2023.02.15.528711; this version posted February 15, 2023. The copyright holder for this preprint (which was not certified by peer review) is the author/funder, who has granted bioRxiv a license to display the preprint in perpetuity. It is made available under aCC-BY 4.0 International license.

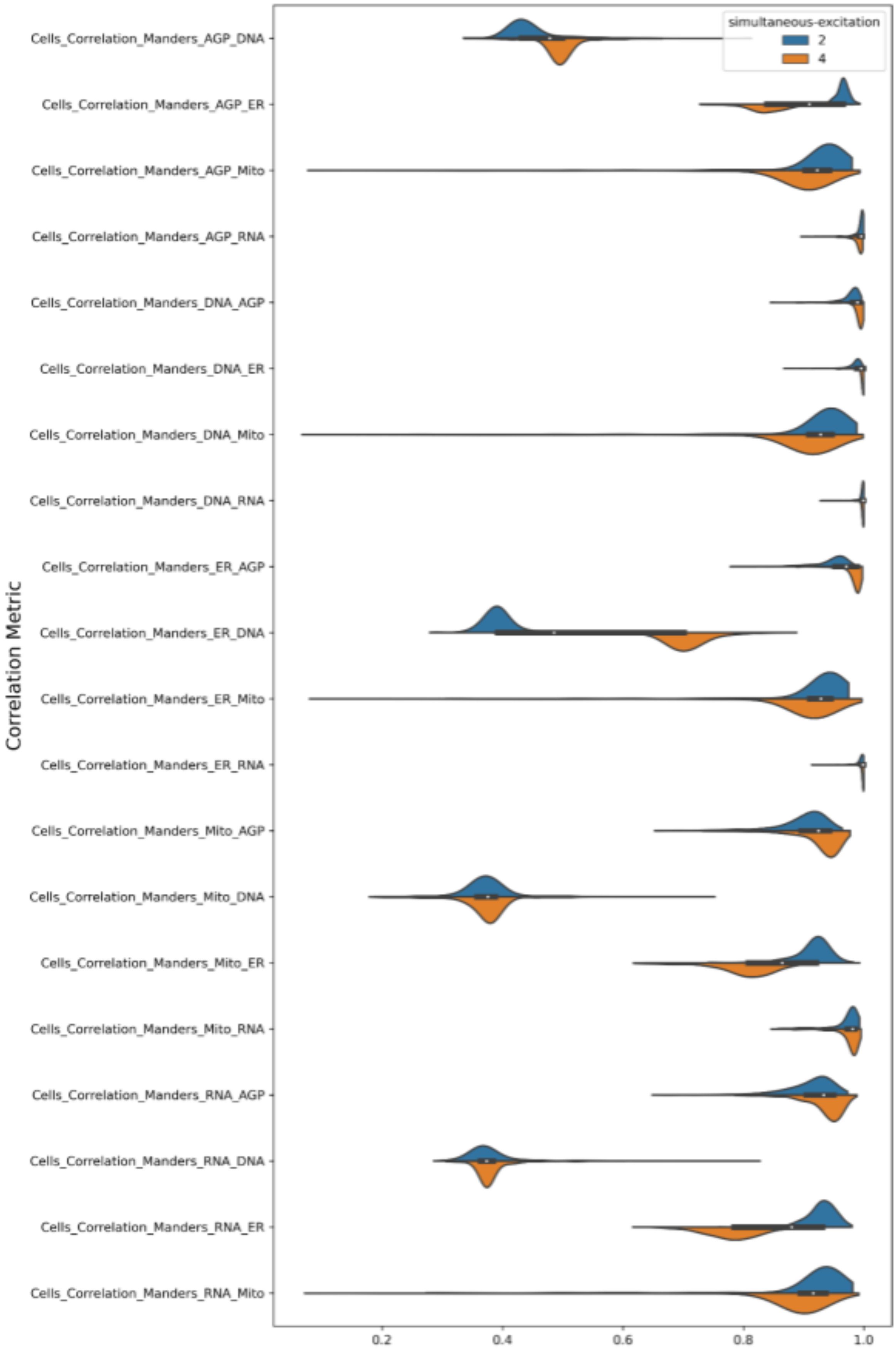

bioRxiv preprint doi: https://doi.org/10.1101/2023.02.15.528711; this version posted February 15, 2023. The copyright holder for this preprint (which was not certified by peer review) is the author/funder, who has granted bioRxiv a license to display the preprint in perpetuity. It is made available under aCC-BY 4.0 International license.

Supplementary figure 4

Unnormalized Manders correlation features within cell objects for 2 or 4x simultaneous excitation profiles. One plate for each condition is presented above. We observe an increase in DNA fluorescence bleedthrough into the ER channel (indicated by the ER_DNA feature above).
